# Development of SMILE-MOM: a metaverse-based support program for single mothers

**DOI:** 10.1186/s12884-025-08099-6

**Published:** 2025-10-02

**Authors:** Minyoung Woo, Sun-Mi Chae

**Affiliations:** 1 Seoul National University College of Nursing, 103 Daehak-Ro, Jongno-Gu, Seoul, Republic of Korea; 2 Seoul National University College of Nursing, The Research Institute of Nursing Science, 103 Daehak-Ro, Jongno-Gu, Seoul, Republic of Korea

**Keywords:** Child, Internet-based intervention, Metaverse, Parenting, Single mothers

## Abstract

**Background:**

Single mothers in South Korea face postpartum challenges that may hinder maternal role development and identity formation. This study aimed to develop SMILE-MOM (Single Moms in the Metaverse for Interaction, Learning, and Encouragement), a metaverse-based support program to assist their transition to motherhood.

**Methods:**

This methodological study employed the first three stages of the Analysis, Design, Development, Implementation, and Evaluation model to develop the program. The analysis phase was informed by an integrative literature review and individual interviews. In the design phase, a theoretical framework guided the creation of program content and the metaverse platform. In the development phase, experts assessed content validity and usability using the Content Validity Index (CVI) and System Usability Scale (SUS), respectively. The final program was refined based on expert feedback.

**Results:**

SMILE-MOM was designed as a 4-week program comprising weekly small-group sessions covering key topics, including maternal preparation and postpartum recovery, infant illness and safety, parenting strategies, and emotional well-being. Each session included hands-on practice activities, such as scenario-based online games, to enhance engagement and facilitate practical learning. The finalized program also included supplementary support services, such as information provision, consultation, and promotion of postpartum exercise, which were embedded within the platform design. The program met content validity (CVI > 0.8) and system usability (SUS = 85.6) standards and was revised based on expert feedback.

**Conclusions:**

This theory-based program supports single mothers in their transition to motherhood through immersive and interactive learning. Although it may enhance maternal role confidence and identity, further evaluation is needed.

**Supplementary Information:**

The online version contains supplementary material available at 10.1186/s12884-025-08099-6.

## Background

The number of women who give birth outside of legal marital relationships has been increasing globally [1, 2]. In many Western countries, including the United States and several European nations, over 30%‒50% of births occur outside of marriage [1]. Although this trend has traditionally been less common in Asia, similar increases have been observed in countries such as Japan and South Korea [1]. In South Korea, for example, the proportion of births registered outside of legal marriage increased from 1.13% in 1981 to 4.72% in 2023 [3]. However, these statistics do not indicate who continues to raise children without a partner. This study focuses on women who both give birth and raise children without a legal or cohabiting partner, whom we define as single mothers.

Despite this trend, single mothers in many Asian societies, including South Korea and Japan, continue to face social expectations and institutional constraints that prioritize traditional marital and familial structures [4, 5]. In contrast to Western countries, where diverse family forms are widely accepted, these mothers often encounter stronger social stigma and reduced access to formal support. These contextual factors contribute to broader structural disadvantages [4, 5].

On an individual level, single mothers experience challenges that interfere with their adjustment to motherhood. Financial strain frequently necessitates balancing employment and caregiving with limited resources [4, 6]. Emotional distress, such as anxiety and depressive symptoms, is especially common during the early postpartum period and is often intensified by inadequate preparation, insufficient support, and uncertainty about the future. Feelings of profound loneliness are also widespread, largely due to a lack of social support and ongoing social stigma [4–7]. Limited opportunities for postpartum recovery and inconsistent access to parenting resources can further undermine both maternal well-being and child development [5, 8]. Although welfare services are available, complicated procedures, limited awareness, and concerns about privacy frequently hinder their use [4, 5].

To address these challenges, many single mothers rely on digital tools and online communities for parenting information and emotional support [5, 9]. Online platforms offer anonymity, convenience, and peer connectivity, features that are particularly valuable for individuals concerned about social stigma [5, 10, 11]. These online tools enhance psychological well-being, parenting knowledge, and caregiving confidence among mothers [11–14].

The emergence of the metaverse has further expanded digital opportunities, particularly since the coronavirus 2019 (COVID-19) pandemic. The metaverse is a virtual environment where users interact in real-time through avatars, enabling social, educational, and cultural engagement across physical and geographic boundaries [15]. Unlike traditional online platforms, it provides users with a heightened sense of presence, facilitates immersive interactions within the virtual environment, and offers affordances that support communication and learning [16, 17]. These qualities make the metaverse particularly suitable for delivering maternal education in ways that feel emotionally safe and socially supportive [10, 18, 19]. For single mothers, metaverse platforms offer a promising alternative to conventional interventions by providing accessible, stigma-free spaces that facilitate flexible learning and peer connection [5, 10, 18]. Prior studies have shown that such environments can enhance health behaviors [20], emotional well-being [10, 13, 18], parenting attitudes [12], and identity formation [19, 21]. Given these advantages, incorporating metaverse-based strategies into maternal support programs may improve their accessibility and effectiveness for single mothers who face substantial social and structural barriers.

Accordingly, this study aimed to develop SMILE-MOM (Single Moms in the Metaverse for Interaction, Learning, and Encouragement), a metaverse-based support program for early motherhood that delivers immersive, evidence-based education and peer interaction to promote maternal adaptation and psychosocial well-being among single mothers in South Korea.

## Methods

**Fig. 1:**
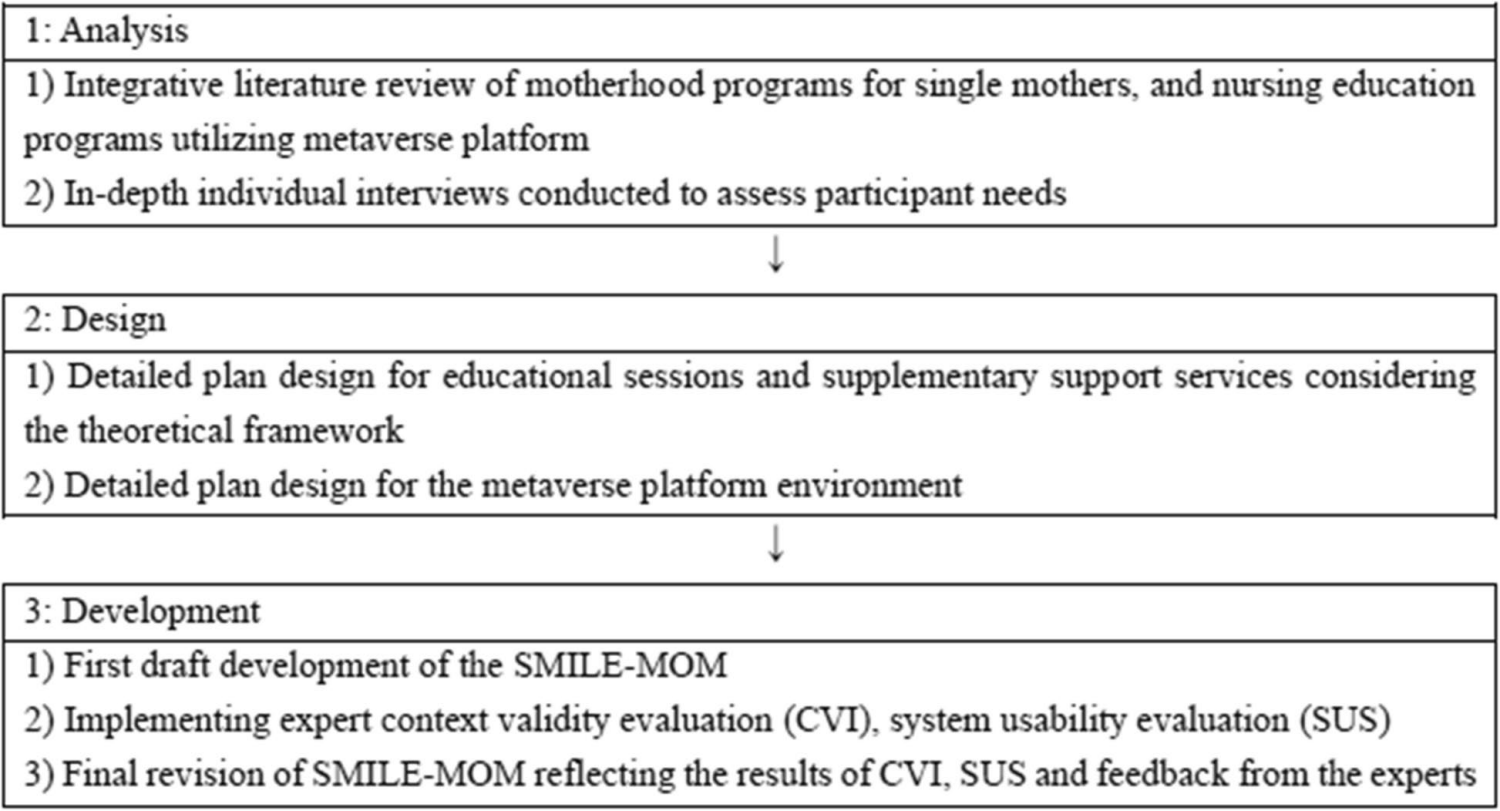
Program development process. Abbreviations: SMILE-MOM, Single Moms in the Metaverse for Interaction, Learning, and Encouragement; CVI, Content Validity Index; SUS, System Usability Scale

This methodological study aimed to develop and validate SMILE-MOM, a metaverse-based support program to assist single mothers in their transition to motherhood. The program was developed in South Korea using the first three phases (Analysis, Design, and Development) of the Seels and Richey ADDIE model, a five-stage instructional design framework consisting of Analysis, Design, Development, Implementation, and Evaluation [22]. These phases guided the systematic identification of needs, integration of theory and evidence, and structured development of platform-based interventions. The Guidance for Reporting Intervention Development Studies in Health Research (GUIDED) checklist was also consulted to enhance clarity and completeness of reporting [23]. Most of the GUIDED elements were incorporated into the development and reporting process, including the context and purpose of the intervention, the target population, theoretical framework, use of evidence, stakeholder involvement, tailoring to the specific needs of single mothers, and iterative revisions based on expert input. An overview of the development process is presented in Fig. 1.

### Needs assessment

#### Integrative literature review

To identify the educational needs of single mothers and determine the core components of a metaverse-based program, an integrative literature review was conducted. The review addressed two main topics: existing motherhood programs for single mothers and nursing education programs utilizing metaverse platforms.

For both topics, literature was retrieved from the Research Information Sharing Service (RISS) and PubMed based on a PICO-SD framework, which guided the inclusion criteria. The population and intervention components were used to define the scope, while comparisons, outcomes, and study designs were not restricted. Studies without comparison groups or clearly defined outcomes were included if they were considered relevant to the purpose of the review. However, studies were excluded if they were purely qualitative or narrative in nature without relevance to program development, or if the full text was unavailable. In PubMed, search terms included both MeSH and natural language keywords, and Boolean operators were used to increase search sensitivity. In RISS, natural language terms were applied. A summary of the search strategy is provided in Table 1.

**Table 1:** Summary of literature review topics and search strategies

Topic	Population and Key search terms	Intervention and Key search terms	Period	Initial search results by database	Selected Studies (*n*)
Motherhood programs	ㆍPopulation: Single mothers ㆍSearch terms: Single parent, Illegitimacy, Unmarried mother, Single mother, Unwed mother, Adolescent mother, Teen mother, Young mother, Juvenile mother	ㆍIntervention: Motherhood programs ㆍSearch terms: Maternal role, Maternal Identity, Parenting, Child rearing, Attachment, Mothering	2010–01-01 to 2024–05-10	ㆍPubMed (420) ㆍRISS (150)	20 (11 domestic, 9 international)
Nursing education programs utilizing metaverse platforms	ㆍPopulation: General population ㆍSearch terms: None	ㆍIntervention: Metaverse-based nursing education ㆍSearch terms: Nursing Education, Program, Intervention, Virtual Reality, Metaverse	2020–01-01 to 2024–05-10	ㆍPubMed (125) ㆍRISS (10)	9 (2 doctoral dissertation, 7 journal articles)

The first topic focused on existing motherhood programs for single mothers. Studies published between 2010 and May 10, 2024 were included to reflect changes in national policy and social attitudes toward single mothers that began around 2010. This period marks a turning point in South Korea, during which institutional support expanded, and community-based programs became more visible [4]. In total, 20 studies, including 11 domestic and nine international publications, were selected based on their relevance to program development. Findings from this review were used to identify essential content domains and delivery methods incorporated into the program design.

The second topic addressed nursing education programs utilizing metaverse platforms. To capture the influence of recent technological advancements and the COVID-19 pandemic, only studies published between 2020 and May 10, 2024 were included. In total, nine studies, including seven journal articles and two doctoral dissertations, were reviewed. Findings from this review informed the selection of platform type, instructional strategies, and spatial configuration for the metaverse-based program.

#### In-depth individual interviews with single mothers

Semi-structured in-depth interviews were conducted with single mothers raising children ≤ 6 years. This criterion was used to focus on early motherhood, a period of heightened caregiving demands and emotional adjustment [24]. The age cutoff also reflects common definitions of early childhood in national parenting support services in South Korea [4]. Participants were eligible if they were aged ≥ 18 years. Individuals with a history of marriage or cohabitation, or with major health conditions that could impair daily functioning or hinder participation in the interview, were excluded. Although the eligibility criteria allowed for the inclusion of adolescent mothers, none were included in the final sample due to cultural barriers and recruitment challenges specific to the South Korean context.

Interviews were conducted between June 14 and July 2, 2024. Participants were recruited through a local single mother support organization using purposive sampling. Eligibility was initially assessed by the organization based on the inclusion criteria and then confirmed by the researcher during direct contact with each referred individual using a brief pre-interview checklist that included questions on major health conditions and marital history. Interviews were held in person, via Zoom, or by telephone, depending on participant preference, and each session lasted approximately 1 h.

The interview guide, which included open-ended questions on motherhood challenges, daily support needs, and perceptions of a metaverse-based educational program, was reviewed by two maternal–child health experts and members of the organization. It was then refined during early interviews with minor adjustments while maintaining consistency in core topics. Written informed consent was obtained from all participants prior to data collection. This study was approved by the Institutional Review Board of Seoul National University (IRB No. 2406/002–005) in accordance with the Declaration of Helsinki.

Thematic analysis was conducted using the six-step method of Braun and Clarke [25]. Rigor was ensured using Sandelowski's criteria of reliability, validity, audibility, and confirmability [26]. To enhance reliability, the findings were reviewed by single mothers’ association.

Findings from the literature review and interviews were synthesized to identify consistent needs and priorities regarding the postpartum adjustment and motherhood experiences of single mothers. The results informed the structure and content of the SMILE-MOM program.

### Program design

The SMILE-MOM program aimed to support single mothers in the early postpartum period by promoting maternal adaptation and psychosocial well-being through an immersive metaverse-based intervention. It was developed by integrating theoretical frameworks with findings from the needs assessment.

The theoretical framework was guided by three complementary frameworks. Mercer’s Becoming a Mother (BAM) model provided the foundation for addressing maternal role confidence and identity [24] and has been widely applied in interventions to enhance maternal sensitivity and readiness [27]. Keller’s ARCS (Attention, Relevance, Confidence, and Satisfaction) model supported motivational strategies [28] and has shown effectiveness in various digital interventions [29]. Picciano’s Multimodal Model for Online Education (MMOE) provided guidance on diverse instructional methods suitable for immersive environments [30]. Social support was also considered a key component, reflecting the interactive nature of metaverse-based learning.

**Fig. 2:**
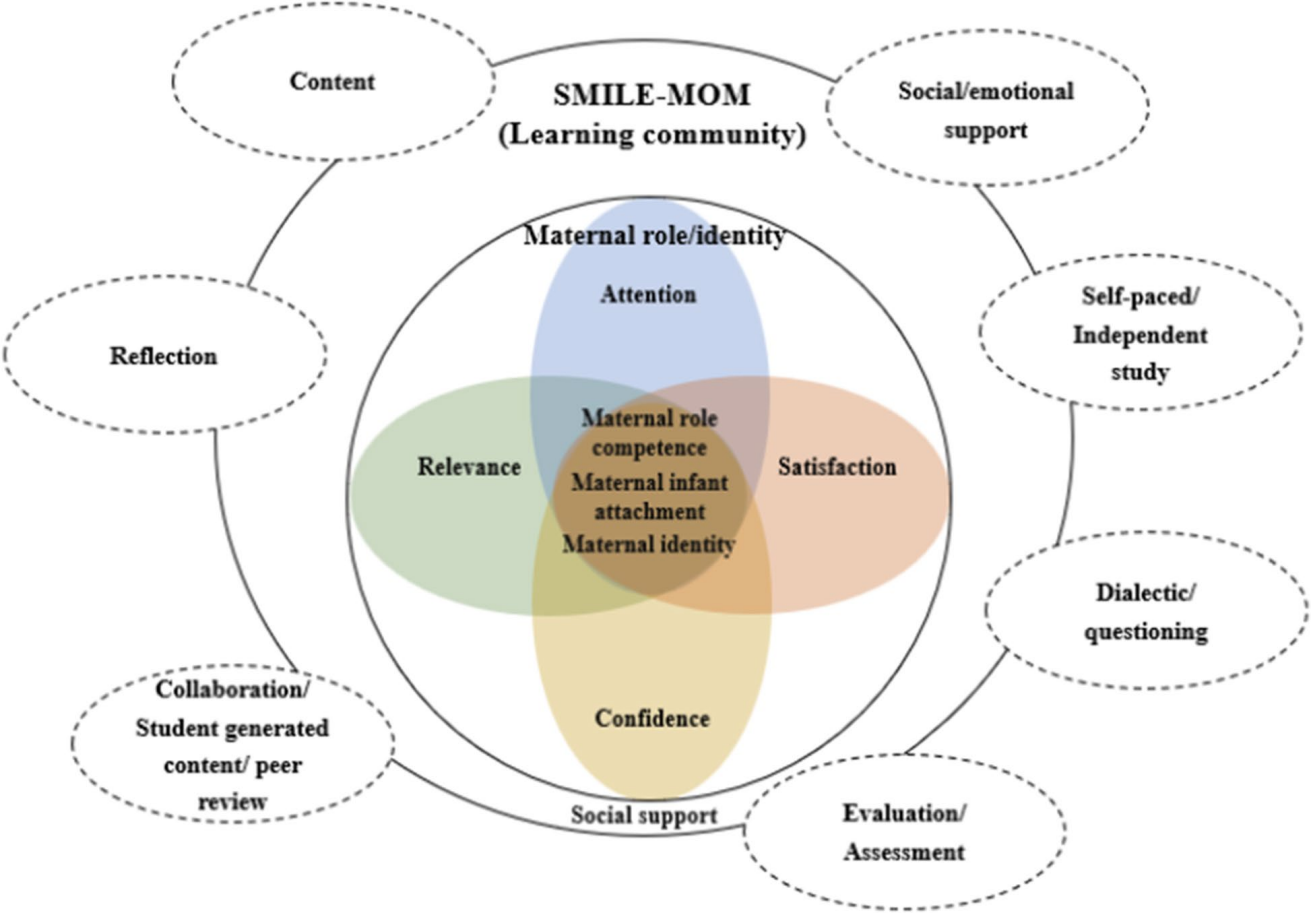
Theoretical framework of the program. Abbreviation: SMILE-MOM, Single Moms in the Metaverse for Interaction, Learning, and Encouragement

These models, applied alongside the needs assessment results, played distinct roles in program design. The design phase aimed at developing a comprehensive instructional plan encompassing learning objectives, content, materials, delivery methods, and the configuration of the metaverse-based environment. The program design was led by the principal researcher. Mercer’s model informed the development of content and instructional materials. Elements of the ARCS and MMOE models were applied to enhance engagement and guide the structure and delivery of the metaverse-based platform. The final integrated framework is illustrated in Fig. 2.

### Program development and validation

Following the design phase, the SMILE-MOM program was developed based on the established instructional plans and platform structure. This phase focused on producing educational materials, implementing the metaverse-based learning environment, and evaluating the content validity and usability of the program through expert review.

#### Development of educational materials

Educational materials were developed for the SMILE-MOM program to align with the instructional plan. The content and worksheets of each session were organized in PowerPoint slides, while additional materials and videos were organized separately. Draft scenarios for scenario-based online games were developed for selected topics.

Content validity was reviewed by a panel of six experts, including professors of maternal-child nursing and experienced clinicians, using the Content Validity Index (CVI). The evaluation followed criteria from previous studies [31, 32] and used a four-point Likert scale (1 = not appropriate, 2 = requires modification, 3 = appropriate with minor revisions, and 4 = highly appropriate and clear). Based on Polit and Beck criteria, an item-level CVI of ≥ 0.78 was deemed acceptable [33]. Expert feedback was reflected in the final revision of the materials.

#### Development of metaverse platform

The finalized materials were transferred to metaverse platform developers to build the prototype of the metaverse-based program. The prototype incorporated both instructional components and interactive features, including scenario-based online games specifically tailored to the objectives of the program.

System usability was evaluated by four nursing experts in extended reality (XR), which encompasses augmented, virtual, and mixed reality technologies, using the Korean-translated System Usability Scale (SUS) [34], with permission from the original developer. The SUS is a widely used tool for evaluating the usability of systems, such as software, mobile apps, and web platforms [34, 35], and consists of ten items, rated on a five-point Likert scale (1 = strongly disagree, 2 = somewhat disagree, 3 = neutral, 4 = somewhat agree, and 5 = strongly agree), resulting in scores ranging from 0 to 100. According to the SUS classification, a score of ≥ 90 is considered excellent, ≥ 80 is good, ≥ 70 is acceptable, ≥ 50 indicates a need for improvement, and < 50 is deemed unacceptable. A previous study reported a Cronbach’s alpha of 0.91 for this tool [36].

## Results

### Needs assessment

#### Integrative literature review

##### Motherhood programs for single mothers

Motherhood programs for single mothers commonly address self-sufficiency, emotional support, and parenting education. Self-sufficiency programs focus on enhancing problem-solving skills, self-efficacy, communication [37–40], community linkage [41], and financial literacy [42]. Emotional support and parenting education are often combined, aiming to promote maternal emotional well-being, recovery [43–49], and attachment-based caregiving [47, 50]. Content areas include baby massage [41], play activities [46], and caregiving skills such as sleep, nutrition, safety, discipline, and child abuse prevention [41, 42, 47, 51].

These programs have demonstrated positive effects on psychosocial outcomes, including reduced anxiety [43–45], depression [42–44, 50, 52], and parenting stress [43, 44, 47, 52, 53], as well as improvements in social support [41, 52], parenting efficacy [37, 38, 45, 46], parenting attitude [42, 47], mother–child attachment [49, 50, 54], and child development [55].

Delivery methods are diverse, including counseling [37, 39, 40, 44, 45], art therapy [38, 49, 50], nature-based interventions [43, 48], digital platforms [43, 48, 53], and home visiting [52, 54, 55]. Most programs are delivered in small groups of four‒10 participants, with session numbers ranging from 5 [56] to 20 [44] and home visit programs extending up to 60 sessions [54, 55]. Sessions typically last 40 min [37, 47] to 2 h [49–51, 56, 57] and are facilitated by nurses, midwives, or community health workers. Although face-to-face delivery is the most common, several programs incorporate online platforms and digital tools such as virtual meeting platforms, educational videos, and discussion-based resources [43, 48].

##### Nursing education programs utilizing the metaverse platform

Among the nine reviewed studies, 67% targeted nurses and nursing students [58–63], 22% focused on patients [64, 65] and 11% involved the public [20]. These programs aimed to overcome the limitations of traditional clinical training by leveraging metaverse platforms to provide immersive, interactive educational environments, particularly in fields such as emergency care [58, 61] and psychiatric nursing [62, 63]. Outcomes reported in the reviewed studies included knowledge, skills, confidence, and satisfaction for nurses and nursing students [58–63], as well as enhanced physical and psychosocial well-being in patients and the public [20, 64, 65].

Three main types of metaverse platforms were identified. Game-based and social networking service-based platforms, such as Roblox and Zepeto, promoted user engagement, social presence, and avatar personalization; however, they lacked instructional control and content management functions, which reduced their applicability in structured educational settings [58, 63, 64]. In contrast, work-based platforms such as Spatial and V-Story offered integrated tools, including real-time conferencing, file sharing, and customizable virtual spaces, making them more suitable for small-group, structured learning [20, 59, 61].

Instructional strategies across studies primarily included role-playing, which facilitated experimental learning in clinical scenarios [58, 59, 61–63]. Other methods, such as group mentoring [60], physical activities [20], game-based interventions [64], and cognitive-behavioral techniques [65], were often combined with supportive technologies, including Padlet, Google Docs, and Zoom to align with multimodal instructional principles for immersive environments.

Common spatial features of the platforms included virtual lecture halls, simulation zones, consultation rooms, and social areas, all of which were tailored to support both instructional activities and peer interaction.

The detailed information extracted and organized from the integrative literature review is available in the Additional file.

#### In-depth individual interviews with single mothers

##### Participant characteristics

Ten single mothers participated in the interviews. Four were in their 20 s, four in their 30 s, and two in their 40 s. All were raising children ≤ 6 years, including one mother with a 3-month-old infant. Two participants had planned pregnancies, whereas eight had unplanned pregnancies. One lived in a single-mother facility, whereas the rest resided independently. Interviews were conducted in person for three participants and remotely for the remaining seven. Participant characteristics are summarized in Table 2.

**Table 2:** General characteristics of in-depth individual interview participants (*N *= 10)

ID	Age (yr)	Child’s Age (yr)	Child’s Sex	Educational attainment	Planned pregnancy	Occupation	Housing type
1	25	3 months	F	College graduate	Unplanned	Company worker	Individual
2	28	2	M	College graduate	Unplanned	Company worker	Individual
3	37	3	M	High school graduate	Unplanned	Housewife	Individual
4	35	5	M	College graduate	Planned	Company worker	Individual
5	46	6	M	High school graduate	Unplanned	Housewife	Individual
6	35	5	F	College graduate	Planned	Housewife	Individual
7	41	4	M	High school graduate	Unplanned	Student	Individual
8	25	4	M	High school graduate	Unplanned	Nursing assistant	Individual
9	21	3	F	High school graduate	Unplanned	Student	Center
10	32	4	M	College graduate	Unplanned	Housewife	Individual

##### Thematic findings

Thematic analysis revealed five major themes that reflected the lived experiences of single mothers during early motherhood: psychological distress, physical difficulties, parenting challenges, social isolation, and structural barriers.

Most participants became single mothers owing to unplanned circumstances and reported substantial challenges that undermined their early motherhood experience, which influenced maternal role confidence and identity formation. Psychological distress was prevalent, with participants describing experiences of emotional instability, loneliness, and depressive symptoms, often triggered by unplanned pregnancies, relationship breakdowns, and the burden of sole caregiving. One participant stated, “Raising a child alone without anyone to share the responsibility is incredibly uncertain. It makes me question everything” (Participant 6). Physical difficulties were also reported, as limited opportunities for postpartum recovery coupled with intensive childcare responsibilities negatively impacted their health. For example, one mother shared, “I struggle to eat regularly, became unwell, and had to visit the hospital frequently” (Participant 1).

In addition to these personal challenges, participants reported difficulties related to parenting, social support, and structural barriers. Many described limited knowledge and experience in childcare, difficulty forming secure attachments with their children, and insufficient access to parenting education or counseling services. This was reflected in statements such as, “Information is scattered and often contradictory, which makes it even more confusing” (Participant 1). Social isolation was a pervasive issue, exacerbated by stigma, strained family relationships, and the absence of reliable support networks. As one participant remarked, “Support only comes by chance. I rarely have any meaningful social interactions” (Participant 10). Structural barriers, including financial hardship, unstable employment, and restricted access to childcare services due to complex administrative procedures and rigid eligibility criteria, further hindered access to support. One mother explained, “Even though I sought help, I was unable to receive it simply because I could not obtain the required certification” (Participant 6).

In this context, participants expressed a strong preference for educational programs that were easily accessible and emotionally supportive. Small-group sessions were viewed favorably for providing peer interaction and reducing feelings of isolation. Digitally delivered or metaverse-based formats were perceived as convenient and less intimidating, especially for those unable to attend in-person services due to time, childcare responsibilities, or stigma. One participant stated, “It would be good to have something I can attend without exposing myself too much, but still feel connected” (Participant 3).

#### Synthesis of literature review and interviews

The synthesis of the integrative literature review and in-depth interviews identified key motherhood-related needs of single mothers during the early postpartum period and potential strategies for effective intervention. The findings highlighted consistent emotional, physical, and structural challenges, as well as needs related to both educational content and delivery methods.

In terms of educational content, both sources emphasized the importance of emotional support, parenting skills such as maternal-infant communication, and maternal preparation. Practical information including postpartum recovery and infant caregiving like managing infant illness and ensuring infant safety, also emerged as a shared priority.

Regarding delivery preferences, studies in literature frequently reported that small-group and interactive formats such as role-play, mentoring, and digital discussions improved engagement, emotional support, and learning outcomes. In parallel, interview participants expressed preference for accessible and emotionally safe programs. They favored small-group settings and digital delivery methods that helped reduce stigma, accommodate scheduling needs, and promote connection with others.

These combined findings suggest that maternal support programs which incorporate socially connected and technology-assisted features, along with relevant and practical content, may be both appropriate and acceptable for addressing the complex needs of single mothers during the early postpartum period.

### Program design

#### Design of educational session and supplementary support services

The SMILE-MOM program was developed to reflect the key needs identified during the needs assessment phase. Its structure includes weekly core group sessions and supplementary support services designed to enhance accessibility, engagement, and ongoing maternal support.

The core group educational sessions focus on four main content areas identified as priorities, including postpartum recovery and maternal adjustment, infant illness and safety, parenting strategies, and emotional well-being. Sessions are delivered once a week over 4 weeks, each lasting 60 min and involving small groups of six‒12 participants. Each session was designed based on the ARCS model of motivation. To capture attention, multimedia materials and reflective prompts were used. Relevance was enhanced by linking content to the live experiences of the participants. Confidence was promoted through interactive activities tailored to single mothers, such as postpartum yoga, scenario-based online games, and peer discussion. Satisfaction was supported by providing meaningful feedback and opportunities for reflection and application. Details of the designed educational session and scenario-based online games are provided in Table 3 and the Additional file.

**Table 3:** Summary of educational session in the SMILE-MOM program

Session (min)	Title (Theme)	Objectives	ARCS	Key Contents	Multimodal Components^a^	Venues in metaverse
1 (60 min)	Stepping into Motherhood (Maternal preparation and postpartum recovery)	1.Reflect on yourself as a mother and recognize your own worth 2.Understand the importance of postpartum health care and practice healthy behaviors	Attention	- Reflecting on early motherhood	① ② ④ ③ ⑤ ⑥ ⑦	Session: Conference room Activity: Exercise room
			Relevance	- Connecting session goals to the motherhood experience of the participants - Reflecting on identity shaped by childhood and family - Exploring ways to seek support related to early challenges - Linking postpartum health management (proper diet and physical activity) to maternal experience - Sharing challenges of motherhood and coping strategies		
			Confidence	- Small group postpartum yoga followed by shared reflection		
			Satisfaction	- Session reflection, attendance rewards, equal access to the session		
2 (60 min)	Providing healthy and safe care for your baby (Infant illness and safety)	1.Learn about the growth and developmental stages of the baby 2.Understand common infant illnesses and essential vaccinations 3.Gain knowledge and skills to ensure the safety of the baby	Attention	- Opening video on infant health	① ② ④ ③ ⑤ ⑥ ⑦	Session: Conference room Activity: House
			Relevance	- Connecting session goals to infant care - Providing practical knowledge on infant health and safety (Infant development and routine checkups, infant diseases and preventive vaccinations, and infant safety management) - Sharing personal experiences related to infant care		
			Confidence	- An 8-item OX quiz - A scenario-based game on infant safety with debriefing		
			Satisfaction	- Session reflection, attendance rewards, equal access to the session		
3 (60 min)	Building attachment with your baby (Parenting strategies)	1.Understand the importance of developing an attachment with your baby 2.Learn to recognize and respond to your baby’s cues 3.Explore ways to build closeness and emotional connection with your baby	Attention	- Opening video on maternal-infant attachment	① ② ④ ③ ⑤ ⑥ ⑦	Session: Conference room Activity: House, Conference room
			Relevance	- Connecting session goals to maternal-infant interaction - Providing knowledge and strategies for building attachment (Maternal-infant attachment, interaction (including play and reaction), and infant cues) - Sharing infant interaction experiences		
			Confidence	- A scenario-based game on infant cues with group debriefing - Small group baby massage		
			Satisfaction	- Session reflection, attendance rewards, equal access to the session		
4 (60 min)	Embracing happiness in motherhood (Emotional well-being)	1.Recognize and understand your own emotions 2.Learn to shift your thoughts in a more positive direction 3.Gain a deeper understanding of yourself as a mother	Attention	- Opening video on emotions and thoughts in motherhood	① ② ④ ⑤ ⑥ ⑦	Session: Conference room Activity: Cafe
			Relevance	- Connecting session goals to emotional experiences - Providing emotion regulation strategies (including postpartum depression) - Sharing personal strengths and emotional coping strategies		
			Confidence	- Mentoring session with senior single mothers to share real-life coping strategies		
			Satisfaction	- Session reflection, attendance rewards, equal access to the session, award ceremony		

^a^ ① Content; ② Social/emotional support; ③ Self-paced/independent study; ④ Dialectic/questioning; ⑤ Evaluation/assessment; ⑥ Collaboration/student generated content/peer review; ⑦ Reflection

In addition to the scheduled group sessions, supplementary support services were provided through the platform to ensure continuity of care and access to resources. These services included information provision, consultation, and promotion of postpartum exercise. Educational materials, additional resources, and participant-requested content were accessible through a virtual library using Padlet, which allowed participants to submit requests and receive uploaded materials accordingly. Private consultations with the researcher or peer mentors were offered via designated spaces or virtual café. A virtual exercise room offered weekly postpartum yoga content, and participants were encouraged to check in daily to maintain engagement.

#### Design of metaverse platform

ZEP (ZEP Co Ltd; https://zep.us/), a two-dimensional browser-based metaverse platform developed in Korea, was selected to deliver the SMILE-MOM program. This platform was chosen for its accessibility via web browsers and mobile devices, Korean-language interface, and high customizability, all of which aligned with the usability needs of single mothers [66].

Dedicated virtual spaces were constructed to support specific session activities and ongoing services. These included a conference room for weekly sessions, virtual house for scenario-based learning activities, library for educational materials, consultation room for private discussions, and café for informal interaction. An exercise room facilitated postpartum physical activity through weekly yoga videos, while a relaxation space promoted mental wellness by offering meditation music and videos.

Engagement features included session reminders sent prior to each meeting and attendance tracking using Google Forms linked to in-platform check-in objects. In addition, a point-based participation reward system was implemented to enhance user motivation and ongoing engagement.

### Program development and validation

#### Educational materials

Educational materials were developed, including PowerPoint slides for the four sessions and scenario-based online game prototypes for Sessions 2 and 3. Each slide set incorporated session objectives, practical activities, discussion prompts, and reflection worksheets.

The draft materials were reviewed by six experts for content validity. CVI for all items exceeded 0.80, indicating strong content validity [33]. Based on expert feedback, the language was simplified throughout the sessions, and visual materials such as postpartum diet images and infant signal facial illustrations were incorporated to enhance comprehension. Furthermore, a genogram activity was introduced in Session 1 to support self-identity through reflection on family of origin. Session 2 was enhanced with practical guidance on child health and caregiving, such as how to respond to common illnesses. To align with the suggestion that self-reflection should precede the discussion of mother–infant attachment in Session 3, relevant activities were incorporated into Session 1. Session 4 was revised to include content on postpartum depression and a self-assessment using the Korean version of the Edinburgh Postnatal Depression Scale (K-EPDS), a validated screening tool for postpartum symptoms [67].

#### Metaverse platform

Using the researcher-designed metaverse platform and finalized educational materials, a prototype of SMILE-MOM was developed in collaboration with a ZEP platform developer. Usability testing by four XR nursing experts yielded individual SUS scores of 80, 80, 95 and 87.5, with an average of 85.625, indicating high usability [36].

**Fig. 3:**
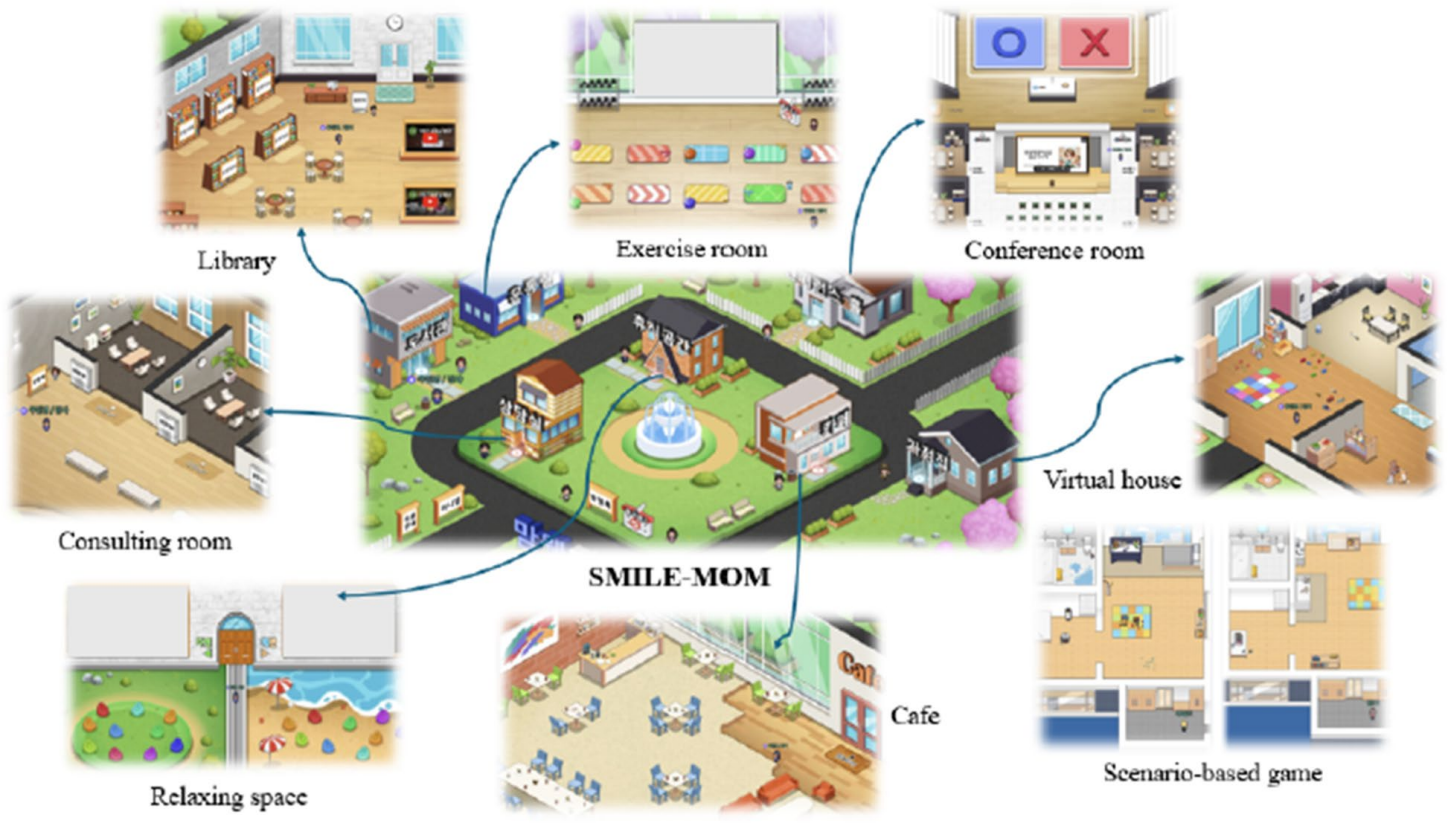
Venues in SMILE-MOM platform. Abbreviation: SMILE-MOM, Single Moms in the Metaverse for Interaction, Learning, and Encouragement

Expert feedback emphasized the need for clearer guidance on using Padlet, discussion boards, and survey items, which were subsequently incorporated into the orientation session of the program. To enhance gameplay flow, spatial descriptions were added to the scenario-based online games in Sessions 2 and 3. The finalized layout of the metaverse environment is shown in Fig. 3.

## Discussion

This study developed SMILE-MOM, a metaverse-based support program for single mothers in South Korea during early motherhood, using the first three stages of the ADDIE model [22]. The design was informed by Mercer’s BAM model [24], Keller’s ARCS model [28], and Picciano’s MMOE model [30] to ensure both theoretical alignment and practical applicability. The program was constructed based on an integrative literature review and in-depth interviews, which identified emotional vulnerability, limited parenting knowledge, and restricted access to social support as key challenges for single mothers [4–7]. SMILE-MOM was designed to address these needs through an immersive digital environment that could be accessed flexibly and used without revealing personal identity. Features such as small-group formats and avatar-based interaction aimed to create emotionally safe spaces, reflecting preferences expressed by participants and characteristics shown in prior research to support stigma-sensitive participation [5, 10, 21].

The program incorporated several elements that distinguish it from conventional motherhood interventions, which often focus on either parenting knowledge or emotional support through face-to-face or home-visiting approaches [37, 47, 52, 54]. Grounded in the BAM model [24, 27], SMILE-MOM integrated identity-oriented and self-reflective components, such as a genogram activity to explore family-of-origin influences and a self-assessment using the K-EPDS [67]. Scenario-based online games on infant safety and infant cues were included to allow participants to engage in realistic caregiving simulations and receive immediate feedback in a risk-free setting. Physical and emotional recovery were addressed through postpartum yoga sessions and a relaxation space embedded within the platform [20].

Motivational and instructional strategies were guided by the ARCS [28, 29] and MMOE [30] models. Reflection activities were deliberately used to sustain motivation, and learner-generated content was supported through a Padlet library where participants could request, share, and co-create resources [62]. The metaverse environment allowed for real-time modification of session activities, enabling adaptation to group needs, and combined synchronous sessions, asynchronous resources, structured mentoring, and informal social spaces [20, 58–66]. By integrating these components, SMILE-MOM provided a flexible, multimodal structure that accommodates diverse participation styles and fosters a sense of community in a stigma-sensitive, accessible format [5, 10, 18, 21].

Despite its strengths, this study has several limitations. First, the effectiveness of SMILE-MOM on maternal outcomes was not evaluated, indicating the need for future research to implement the intervention and verify its effects. Second, although the program was conducted over four weeks, this period corresponds to the critical postpartum stages described in Mercer’s BAM model [24], the second stage (Acquaintance, Practice, and Physical Restoration) and the third stage (Approaching Normalization). These stages involve recognizing and adapting to physical and emotional changes after childbirth and forming maternal identity, which align with the program’s objectives and timing. Nevertheless, maternal role development is a continuous process, and sustained support beyond this initial period is essential. Future studies should consider longer-term interventions or follow-up modules to address the ongoing nature of maternal adaptation. Given SMILE-MOM’s online platform, which minimizes temporal and spatial constraints, it has strong potential to evolve into a continuous support system that enables long-term engagement.

Third, although the platform was designed for accessibility, participants may still encounter technical challenges related to device performance, digital literacy, and internet connectivity. Such barriers are well-documented in the context of digital health equity [68, 69], underscoring the importance of user-centered design and adequate implementation support to reduce disparities in access and engagement. Finally, ZEP, the 2D metaverse platform used in this study, may offer lower levels of immersion compared to 3D virtual reality systems. To enhance long-term engagement, future versions of SMILE-MOM may incorporate adaptive content updates, gamification elements, and asynchronous participation formats.

Nevertheless, SMILE-MOM represents an innovative application of theory-driven digital intervention in maternal nursing. By integrating empirical evidence, user input, and educational theory, the program provides a flexible and stigma-sensitive support structure for single mothers navigating the early postpartum period. This study underscores the potential of metaverse-based learning to expand maternal care beyond clinical boundaries and offers a replicable model for digital health interventions targeting underserved parenting populations. It also highlights the evolving role of nurses as designers and facilitators of theory-based, technology-enhanced interventions, demonstrating how nursing practice can be expanded to meet emerging maternal health needs in virtual environments.

## Conclusions

This study developed and validated SMILE-MOM, a metaverse-based support program for single mothers in early motherhood. The program was systematically designed using theoretical frameworks and empirical findings to address emotional and physical challenges, strengthen parenting skills, and reduce social isolation. It consists of four structured weekly sessions with supplementary support services, organized to be adaptable to an immersive, interactive environment that can foster engagement and peer connection.

The study did not include outcome evaluation, so the effects of the program on maternal outcomes remain to be determined. Future research should implement the intervention and assess its effectiveness using mixed-method or experimental designs. Expanding the program into open-access or asynchronous formats may further enhance accessibility, support sustained engagement, and extend its reach to a broader population of single mothers.

## Supplementary Information

Supplementary Material 1.

## Data Availability

The datasets generated and/or analysed during the current study are not publicly available because it involves participants’ personal information. However, additional data regarding the SMILE-MOM program are available from the corresponding author on reasonable request.

## Abbreviations

SMILE-MOM: Single Moms in the Metaverse for Interaction, Learning, and Encouragement
ADDIE: Analysis, Design, Development, Implementation, and Evaluation
CVI: Content Validity Index
SUS: System Usability Scale
COVID-19: Coronavirus 2019
RISS: Research Information Sharing Service
BAM: Becoming A Mother
ARCS: Attention, Relevance, Confidence, Satisfaction
MMOE: Multimodal Model for Online Education
XR: Extended Reality
K-EPDS: Korean version of the Edinburgh Postnatal Depression Scale
