# Supplementary material for: Development of SMILE-MOM: a metaverse-based support program for single mothers
Source: BMC Pregnancy Childbirth. 2025 Oct 2;25:1003. doi: 10.1186/s12884-025-08099-6 (PMC12492609; doi:10.1186/s12884-025-08099-6)
Supplement: Supplementary file 1 — Supplementary Material 1. [file 12884_2025_8099_MOESM1_ESM.pdf]

Additional File

<1> Review of motherhood program for single mothers

Author (Year, Nation)	Intervention theme	Participants (Size, n)	Study design	Intervention Characteristics: Intervention Group (IG)	Control group (CG)	Methods	Type (Group/ Individ-ual)	Outcome variables: Measure time	Results
Kwon BH, Kim GS (2015, Korea)	Art therapy (Doll making)	Single mothers living in support center (age: 18–21 years) (IG: 7)	SGPP (+qualit-ative mixed)	A total of 12 sessions, each 1.5–2 h, Two–three sessions per week (total 1 month) (1–4: attachment experience, 5–10: self-caring, 11–12: wrap up)	-	Art supplies	Group (seven members (mem))	Pre and post	Quantitative<br>1. Significant<br>- Depression<br>2. Non-significant<br>- Attachment<br><br>Qualitative<br>● Emotional: affection↑, attachment↑<br>● Psychological: depression↓
Kim MH, Park SO (2013, Korea)	Art therapy (Creative problem solving)	Single mothers living in support center (age: 16–34 years) (IG:8, CG:8)	QED	A total of 16 sessions, each 1.5 h, Two sessions per week (total 2 months (1–3: visualization and goal discovery, 4–6: fact identification, 7–13: problem identification, 14–16: solution discovery)	none	Art supplies	Group (eight mem)	Pre and post	Quantitative<br>1. Significant<br>- Self-efficacy, Problem solving
Kim SY (2014, Korea)	Group counseling	Single mothers living in support center (IG:8)	Method-ological	A total of 10 sessions (1–2: understanding self and others; 3–7: conflict resolution, communication facilitation; 8–9: self-esteem; 10: self-determination)	-	Counseling, handout, game	Group (eight mem)	-	No outcome measured
Kim JU, Kim YK (2018, Korea)	Happiness enhanceme-nt program	Single mothers living in support center (age: 20 s–50 s) (IG:10, CG:10)	QED	A total of eight sessions, each 1-1.5 h, two sessions per week (1: orientation; 2: happiness; 3: personality strengths; 4: positivism; 5: valor; 6: thankfulness; 7: forgiveness; 8: wrap-up)	none	Counseling, handout, video, game	Group (10 mem)	Pre and post	Quantitative<br>1. Significant<br>- Resilience, anxiety, self-efficacy
Park IT, Oh WO (2020, Korea)	Child abuse prevention	Single mothers living outside support center (age: 19 years↑) (IG: 7)	SGPP (+qualit-ative mixed)	A total of 10 sessions, each 0.7 h (40 min), prenatal twice a week for 2 weeks, postpartum twice a week for 3 weeks (prenatal 1–4: parental preparation, emotional support, maternal health, family, and friend support; postpartum 5––10: maternal infant attachment, child development and caregiving, emotional regulation, prevention of child abuse, family environment, and social support)	none	Video, handout, counseling	Individual	Pre and post (antepa-rtum, postpar-tum separta-tely)	Quantitative<br>1. Significant<br>- Parenting attitude, maternal-child interaction<br>2. Non-significant<br>- Resilience, parenting stress, fetal attachment, neonatal perception, child abuse potential<br><br>Qualitative<br>● Program usability: positive

Eom Y et al. (2021, Korea)	Emotion regulation program	Single mothers living in support center (age: 10 s–20 s) (IG: 11)	SGPP (+qualitative mixed)	A total of 20 sessions, weekly, each 1 h (1: orientation, pre-test; 2–4: emotion education; 5–10: emotion regulation technique; 11–13: cognitive reconstruction; 14–16: problem solving technique; 17–19: relapse prevention; 20: wrap up, post-test)	-	Handout, counseling	Individual	Pre and post	Quantitative<br>1. Significant<br>- Anxiety, depression, parenting stress<br><br>Qualitative<br>● Emotional↑, cognitive↑, behavioral↑, relapse prevention↑
Yoon MR, Choi SN (2013, Korea)	Group art therapy	Single mothers living in support center 14–27 years) (IG:5, CG:5)	QED (+qualitative mixed)	A total of eight sessions, each 1.5—2 h, two sessions every week, for 1 month (1–2: understanding prenatal education, self-awareness of the pregnant person, and perception of the relationship with the fetus; 3–6: the process of exploring one's positive self-image, navigating life changes, fostering hope for the future; 7–8: accepting oneself as they are, preparing gifts for the unborn baby, thinking positively, and providing empathy and support among peers)	None	Art supplies	Group	Pre and post	Quantitative<br>1. Significant<br>- Psychological wellness<br>2. Non-significant<br>- Maternal-fetal attachment<br><br>Qualitative<br>● Behavioral↑
Yoon S, Chang J (2021, Korea)	Group counseling program	Single mothers and fathers	Methodological	A total of 10 sessions, each 1.7 h (100 min) (1: open-heartedness, 2: goal setting, 3–6: group work based on indicators, stress coping strategies by Myers-Briggs Type Indicator (MBTI), 7–9: establishing relationships with children, parents, and oneself, 10: strengthening independence, sharing experiences)	-	Handout, counseling	Group	-	No outcome measured
Lee SJ, Han Y (2019, Korea)	Group art Therapy	Single mothers living in support center (age 19 years↑) and their child (age 12 months–5 years) (IG:5, CG:5)	QED	A total of 10 sessions (group art therapy 1 h + Child-Parent Relationship Therapy (CPRT) 1.5 h (group parenting education 0.5 h, individual supervision (three–nine sessions) 0.5 h, individual special play time (three–nine sessions) 0.5 h)) weekly for 10 weeks<br>(1 (one–three sessions): Introspection (CPRT: encouragement to prepare for and initiate special playtime at home);<br>2 (four–seven sessions): Inner acceptance (CPRT: improving empathetic interaction skills through learning play therapy techniques and practicing them);<br>3 (eight–10 sessions): Inner growth (CPRT: strengthening play therapy techniques and providing support to apply these skills, enabling their generalization not only in special	none	Art supplies, handout, counseling	Group, Individual	Pre and post	Quantitative<br>1. Significant<br>- Guilt, parenting efficacy, mother-child relationship

				playtimes but also in daily life)					
Supporting team for single-parent family (2016, Korea)	Building psychological boundary program	Single mothers (age: 19 years↓)	Methodological	A total of five sessions, each 2 h (1: contain; 2: add up; 3: take care of; 4: take heart; 5: step forward)	-	Handout, art supplies, counseling	Group (four–10 mem)	-	No outcome measured
Ji SY (2012, Korea)	Pre-parent education program	Single mothers living in support center 17–24 years) (IG:12,CG:12)	QED	A total of eight sessions, each 2 h, once or twice a week, total 4–8 weeks. (1–2: understanding mother herself; 3–5: decreasing learned helplessness; 6–8: performing parent role)	none	Video, handout	Group (five–seven mem)	Pre and post	Quantitative<br>1. Significant<br>- Helplessness, Satisfaction with the parenting role
Kang S et al. (2020, Korea)	Preparation for independence program	Single mothers living in support center 4 years) (IG:6, CG:6)	QED	A total of 10 sessions, twice a week for 5 weeks, each 0.7 h (45 min) (1: mind opening, 2: goal setting, 3–8: establishing relationships with children/others/oneself, 9: building foundation of independence, 10: sharing group counseling experiences)	none	Handout, counseling	Group	Pre, Post and 2 weeks later	Quantitative<br>1. Significant<br>- Parenting efficacy, independence will, emotional independence<br>2. Non-significant<br>- Independence level, self-esteem
Bang KS et al. (2023, Korea)	Natural wellness group program	Single mothers (age: 18 years↑) ,children (age: 0–6 years) (IG:18, CG:19)	QED (+qualitative mixed)	A total of eight sessions, each 1.5 h, weekly interactive real-time video platform (1–6: reduce anxiety and depression and improve self-esteem to promote health; 7: promote health and lower parenting stress; 8: reduce anxiety and parenting stress)	none	Virtual meeting platform, online chat room, text message, mail, forest treatment supplements	Group (four–seven men)	Pre and post	Quantitative<br>1. Significant<br>- Mental health, depression, self-esteem<br>2. Non-significant<br>- Physical health, anxiety, parenting stress<br><br>Qualitative<br>● Physical (Relaxed, energized)<br>● Psychological (Anxiety↓, depression↓, positive feeling)
Alarcao FSP et al. (2021, Brazil)	Nurse Home visiting program	Adolescent mothers 14–19 years, 1st trimester preg) (IG:40, CG:40)	RCT	A total of 60–62 visiting (1st trimester–24mo of child) (weekly visit: first/last month of pregnancy/puerperium; biweekly visit: gestation/2–20 months of child's age; monthly visit: 21–24 months of child's age)	Usual care	Counseling	Individual	Infant 6 months, 12 months	Quantitative<br>1. Significant<br>- Infant attachment (12 months)<br>2. Non-significant<br>- Maternal attachment (12 months), infant neural signal (6 months)<br><br>Correlational<br>● Small neural signal amplitudes associate with better social behavior and attachment at 12 months
Barnes J et al. (2017, UK)	Group Family Nurse Partnership (gFNP)	Expectant mothers 24 years↓) (IG:57, CG:44)	RCT	A total of 44 sessions (1st trimester–12 months of child), each 2 h (14 sessions about pregnancy, 30	Usual care	Counseling	Group	Pregnancy, Infant 2 months, 6 months,	Quantitative<br>1. Non-significant<br>- Parenting attitudes, interaction quality

				sessions about infancy; interaction and often hands-on activities)				12 months	Economic<br>● No positive economic effect
Fatori D et al. (2021, Brazil)	Nurse Home visiting program	Adolescent mothers<br>14–19 years)<br>(IG:40, CG:40)	RCT	Total 66 visiting (1st trimester–24months of child)<br>(weekly visit: first/last month of pregnancy/puerperium; biweekly visit: gestation/2–20 months of child's age; monthly visit: 21-24 months of child's age)	Usual care	Counseling	Individ-ual	Infant birth,<br>3 months,<br>6 months,<br>12 months,<br>24 months	Quantitative<br>1. Significant<br>- Language development, maternal responsivity, home stimulation<br>2. Non-significant<br>- Electroencephalography response, physical growth
Sachs AL et al. (2022, USA)	Meeting in Nature Together Program	Pregnant and parenting teenagers in the charter school<br>(age: 14–19 years)<br>(IG1:8, IG2:8)	SGPP (+qualit-ative mixed)	Fall 2020(Session 1), Spring 2021(Session 2), each 1 h, weekly session<br>Session 1: A total of eight sessions through online<br>Session 2: Modified eight sessions (in person added) + three park walk sessions<br>(enjoying nature through photography, walking, scheduling time and support relatedness and social connectedness)	-	Phone app (iNaturalist), physical activity, photography, Counseling, Google classroom, Google meet, peer-group supported nature-based activities	Group	Pre and post	Quantitative<br>1. Mixed significant<br>- session 1: loneliness↓, session 2: loneliness↑<br><br>Qualitative<br>● Emotional/psychological↑, social connection↑, nature connection↑, program acceptability↑
Hudson DB et al. (2012, USA)	Internet based social support intervention	Single adolescent mothers (African American, age 16–21 years,<br>1 week postpartum)<br>(IG:15, CG:19)	PLED	New Mothers Network (NMN) accessible for 6 months<br>(1: electronic library about caring mother themselves and infants; 2: asynchronous discussion forum with nurses and one another; 3: e-mail to nurses)	Usual care	Broad band connection	Group	Postpar-tum,<br>1 week,<br>6 weeks,<br>3 months,<br>6 months	Quantitative<br>1. Significant<br>- Self-esteem<br>2. Non-significant<br>- Depressive symptoms, loneliness, stress, parenting competence, parenting satisfaction, healthcare utilization outcomes (clinic visits, hospitalizations, emergency room visits)
Cohen D et al. (2011, UK)	Midwifery support	Pregnant teenage women	RCT	Usual midwifery care plus psychosocial support (at a time suited for client; focused on antenatal period; 1: training such as child protection, parenting, sexual health promotion, baby massage; 2: links with other agency staffs such as Sure Start; 3: creation of favorable environment for behavioral change; 4: introduction of YoungMumsProject services to improve lifestyle)	Usual care	Counseling	Individ-ual	Pre and post<br>(4 weeks postpar-tum)	Quantitative<br>1. Non-significant<br>- Self-esteem, physical activity, social support, smoking behavior, uptake of other services (but IG greater use of other services than CG)
Cox JE et al. (2019, USA)	Parenting and life skills	Mothers<br>(age: 19 years↓),<br>infants<br>12 months↓)<br>(IG:72,	RCT	Teen-tot (Teenage-Toddler) services+5 sessions of parenting and life skills (each session 1 h long, 1:1 interactive modules;<br>1. Child development and discipline; 2: safety;	Teen-tot service only	Counseling	Individ-ual	Pre and Infant 12 months,<br>24 months,<br>36	Quantitative<br>1. Significant<br>- Maternal self-esteem, repeat pregnancy (within 36 months)<br>2. Non-significant<br>- Parenting attitudes related to child

		CG:68)		3: Budgeting and bank account; 4: job and education, 5: healthy living)				months	maltreatment risk, maternal depression, life skills
Samanka-sikorn W et al. (2016, USA)	Community Health Worker (CHW) Home visiting program	Pregnant teen mothers (IG:83, CG:66)	RCT	Resource Mothers Program home visiting (twice a month during preg, monthly up to 1 yr postpartum; social support, role modeling, screening, health education, referrals; none of the CHWs are nurse)	Tele-phone support	Counseling	Individual	Pre and 3 months postpar-tum	Quantitative<br>1. Significant<br>- Depression, self-esteem<br>2. Non-significant<br>- Maternal stress, social support

PLED: Pilot Longitudinal Experimental Design; QED: Quasi-Experimental Design; RCT: Randomized Controlled Trial; SGPP: Single Group Pretest Posttest

< 2 > Review of nursing education program using metaverse platform

Author (Year, Nation, paper type)	Intervention theme (methods)	Participants (Size, n)	Study design	Intervention Characteristics				Outcome variables	
				Metaverse platform / Other materials	Type (Group / Individual)	Intervention Group (IG)	Control group (CG)	Measure time	Results
Kim J (2023, South Korea, Doctoral dissertation)	Conflict management among nurses (simulation)	Senior grade nursing students (IG:22, CG:21)	RCT (+qualitative mixed)	V-story (Virbela) (lecture: conference hall; team activity: private area with round table; role play: conference hall stage)	Group (four members (mem)/team, two teams/one group)	Total 3 sessions, each 1.5 h (1: lecture and team activity; 2–3: prebriefing, role play, debriefing)	Online video lecture (conflict related learning content in nursing management)	Pre, post and 6 weeks follow up	Quantitative<br>1. Significant<br>- Conflict efficacy, interpersonal communication<br>2. Non-significant<br>- Satisfaction, self-confidence<br><br>Qualitative<br>● Learning experience
Ryu EJ (2024, South Korea, Doctoral dissertation)	Schizophrenia nursing (simulation)	Nursing students (IG: 15)	SGPP (+qualitative mixed)	Spot / Padlet, Google jamboard (lecture: lecture room for each session; debriefing and team discussion: private team room and Google jamboard; role play: virtual room for each patient scenario; entire debriefing: debriefing room; wrapping up quiz: quiz zone)	Group (five mem/team)	A total of four sessions, each 2 h one session per week (1: 1.5 h, schizophrenia lecture, adapting metaverse; 2: 1 h, understanding patients, nursing process; 3: 1 h scenario, 1 h debriefing, scenario development, patient journey map; 4: 1 h role play, 0.5 h debriefing)	-	Pre and post	Quantitative<br>1. Significant<br>- Knowledge, learning presence, learning flow, learning satisfaction<br>2. Non-significant<br>- Learning confidence<br><br>Qualitative<br>● Program quality evaluation both instructor and students
Kang M, Moon SH (2023, South Korea, published journal)	Emergency nursing (simulation)	Junior grade nursing students (IG: 54)	SGPP	Zepeto (Emergency room is designed by proficient team skilled in BuildIt and Photoshop)	Group (five–six members/team)	A total of three sessions (1: 2 h, program orientation, scenario development; 2: 0.3 h adapting metaverse, 0.7 h role play(simulation); 3: 0.5 h, debriefing)	-	Pre and post	Quantitative<br>1. Significant<br>- Communication sub-factors: connecting, rapport building<br>2. Non-significant<br>- Overall communication, problem-solving process, learning self-efficacy
Moon SH (2023, South Korea, published journal)	Emergency nursing (simulation)	Nursing students (IG: 34, CG: 38)	NCGP	V-story (Virbela) (lecture: lecture hall; team activity: seminar room; role play: conference hall stage)	Group (five–six members/team)	A total of three sessions (1: 1 h, metaverse orientation, adapting metaverse; 2: 3 h, team activity, case analysis, scenario development; 3: 1 h, role play)	None (Completed only adult nursing courses)	Post only	Quantitative<br>1. Significant<br>- Clinical skill sub-factors: communication, nursing process<br>2. Non-significant<br>- Overall clinical skill, problem-solving efficacy, learning satisfaction
Lee JH et al (2023, South	Social interaction	High Functi-	PRCT	Roblox / ZOOM (orientation with PPT: ZOOM	Group	A total of four sessions, each 1 h, one session per	None	Pre and post	Quantitative<br>1. Significant

Korea, published journal)	program for Autism Spectrum Disorder (ASD) children (sports game)	oning ASD children (IG: 9, CG: 6)		/ game & teaching: Roblox soccer field)		week; Each session with different goals; Each session consisted of theoretical classes, practice of metaverse, homework, and feedback; Conducted by one clinical psychologist and two nurses; program is evaluated by ASD expert			- Social responsiveness, mindfulness, adaptive behavior (some areas), caregiver psychological well-being (some aspects)<br>2. Non-significant<br>- Core ASD symptoms, emotional/behavioral problems, depression, anxiety
Kim Y, Kim MY (2023, South Korea, published journal)	Career mentoring	Nursing Students [38] & Mentors (Registe-red Nurse, 8)	SGPP (+qualit-ative mixed)	Metaforest	Group (eight members max/session)	Select two amongst total eight sessions (Group mentoring, each different career, 60 min)	-	Pre (Zoom) and Post (metave-rse)	Quantitative<br>1. Significant<br>- Career self-efficacy, program satisfaction<br>- Metaverse > Zoom (mentees): immersion, togetherness, interactivity, emotional expression<br>- Metaverse > zoom (mentors): emotional expression<br>● Zoom > Metaverse: fatigue<br><br>Qualitative<br>● Satisfied with candid, realistic communication and program functions, but expecting more optimized program
Yang SY, Kang MK (2023, South Korea, published journal)	Schizoph-renia nursing (simulati-on)	Junior grade nursing students (IG: 29, CG: 29)	NCGPP	ZEPETO / Google Docs, online learning management system (OLMS) (orientation: hospital, and conference rooms; online lecture: OLMS; simulation: hospital; team activity: Google Docs; briefing: conference rooms)	Group (four–five members/team)	A total of three sessions (1: 2.5 h prebriefing, orientation 50 min, online lecture 100 min; 2: 1.6 h, 2 Simulations each 50 min; 3: debriefing 100 min)	Only online lecture	Pre and post	Quantitative<br>1. Significant<br>- Knowledge, critical thinking, communication<br>2. Non-significant<br>- Learning self-efficacy, learning satisfaction, confidence
Robinson N et al. (2023, USA, published journal)	Substance Use Disorder (SUD) treatment (Cognitive behavioral therapy,	Recover-ing individu-als from SUD (IG: 48)	PSGPP (+qualit-ative mixed)	Innerworld (used Oculus Quest Virtual Reality (VR) headset and handheld controllers)	Group	As many as possible (no mention of entire intervention period), each session 60 min, daily CBT (coaches set agendas for each group, adjusting the contents for each groups' needs) and	-	Pre and post	Quantitative<br>1. Significant<br>- Positive affect<br>2. Non-significant<br>- Negative affect, online social support<br><br>Qualitative

	CBT)					weekly group consultation			● Program satisfaction: satisfied with connection and content, but usability and fatigue need improvement
Mizuta R et al. (2024, Japan, published journal)	Physical activity	Young adults (IG:16, CG1:16, CG2:16)	RCT with three groups	Spatial / YouTube (IG: exercise room in Spatial; CG1: YouTube video watching)	Group	A total of eight videos for 8 weeks (one video uploaded in Spatial for each week); able to watch video at any time	CG1: total 8 videos for 8 weeks (one video uploaded in YouTube for each week); able to watch video at any time<br>CG2: none	Pre and post	Quantitative<br>1. Significant<br>- Physical activity<br>2. Non-significant<br>- Well-being, locomotive function, social capital

NCGPP: Non-equivalent Control Group Pretest-Posttest; NCGP: Non-equivalent Control Group Posttest-only; PRCT: Pilot Randomized Controlled Trial; PSGPP: Pilot Single Group Pretest Posttest; RCT: Randomized controlled trial; SGPP: Single group pretest posttest

〈 3 〉 Contents of educational session in SMILE-MOM program

Session No. 1

**Title**	**Stepping into Motherhood**			
**Objectives**	1. Reflect on yourself as a mother and recognize your own worth<br>2. Understand the importance of postpartum health care and practice healthy behaviors			
**Preparation materials**	1. Paper educational materials (contents and worksheet)<br>2. Postpartum yoga video		**Running time**	60 min
**Contents and components**	**ARCS categories**	**Contents**	**Multimodal model components**	**Venues in the metaverse**
	**Attention**	**Perceptual arousal, Inquiry arousal, Variability**<br>≫Reflecting on their thoughts and feelings when they first became mothers	≫Dialectic/questioning<br>≫Collaboration/student generated content/peer review	Conference room
	**Relevance**	**Goal orientation**<br>≫Confirming objectives for this session and relating them to participants' current motherhood status<br>**Motive matching**<br>≫Sharing reflections on self-image and maternal identity in relation to childhood and family influences<br>≫Exploring ways to seek support from others in addressing related past challenges<br>≫Relating postpartum health management, such as appropriate nutrition and physical activity, to current identity and experiences of mothers<br>**Familiarity**<br>≫Sharing experiences of the challenges of motherhood and strategies for	≫Content<br>≫Social/emotional support<br>≫Dialectic/questioning<br>≫Collaboration/student generated content/peer review	Conference room

		overcoming them		
	**Confidence**	**Presenting objectives of the session**<br>≫Explaining the importance of postpartum health management, including proper diet and physical activity, for recovery and well-being<br>**Success opportunities**<br>≫Group exercise: Performing postpartum yoga in small groups<br>**Personal controls**<br>≫Taking breaks during activities if needed<br>≫Engaging with interactive features, such as avatar movement		Exercise room
	**Satisfaction**	**Natural consequences**<br>≫Reflecting on the session experience<br>**Positive consequences**<br>≫Receiving attendance-based incentives<br>**Equity**<br>≫Ensuring equal access to the educational content and activities for all participants	≫Self-evaluation/assessment<br>≫Collaboration/student generated content/peer review<br>≫Reflection	None
**Supplemental Materials**	1. 2024 Single-parent family welfare service comprehensive guide: Booklet<br>2. Successful breastfeeding and breast care: Internet link<br>3. Postpartum yoga exercise video: Internet link		≫Content<br>≫Self-paced/independent study	
**Note**	1. Send a text message encouraging participation the day before educational session.<br>2. Notice regarding attendance point: entering the platform (1 point/day), using the exercise room (1 point/day), participating educational session (1 point)<br>3. Notice that the postpartum yoga video of the exercise room will be uploaded as a different video every week<br>4. Set 2nd session class schedule and notice participants 2nd session materials will be uploaded in library			

Session No. 2

<table>
<tr><td>Title</td><td colspan="4">Providing healthy and safe care for your baby</td></tr>
<tr><td>Objectives</td><td colspan="4">1. Learn about the growth and developmental stages of the baby
2. Understand common infant illnesses and essential vaccinations
3. Gain knowledge and skills to ensure the safety of the baby</td></tr>
<tr><td>Preparation materials</td><td colspan="2">1. Paper educational materials (contents and worksheet)
2. Opening video
3. OX quiz and related descriptions
4. Scenario-based games and related descriptions including debriefing questions</td><td>Running time</td><td>60 min</td></tr>
<tr><td rowspan="3">Contents and components</td><td>ARCS categories</td><td>Contents</td><td>Multimodal model components</td><td>Venues in the metaverse</td></tr>
<tr><td>Attention</td><td>Perceptual arousal, Inquiry arousal, Variability
≫Showing an opening video related to infant health</td><td>≫Content</td><td>Conference room</td></tr>
<tr><td>Relevance</td><td>Goal orientation
≫Confirming the session objectives and aligning them with participants current experiences related to infant care, health, and safety
Motive matching
≫Providing practical knowledge and strategies to enhance maternal role confidence (such as infant development, health check-ups, common illnesses, vaccinations, and safety management)
Familiarity
≫Sharing personal experiences of managing infant illness or coping with safety-related incidents</td><td>≫Content
≫Social/emotional support
≫Dialectic/questioning
≫Collaboration/student generated content/peer review</td><td>Conference room</td></tr>
</table>

	Confidence	**Presenting objectives of the session**<br>≫Explaining the importance of understanding maternal roles and infant care prior to participating in the OX quiz and scenario-based activity<br>**Success opportunities**<br>≫OX quiz: Completing an 8-item OX quiz based on session content<br>≫Scenario-based game (Infant safety): Participating in a scenario-based game identifying and removing infant safety threats, followed by group debriefing<br>**Personal controls**<br>≫Taking breaks during activities if needed<br>≫Engaging with interactive features, such as avatar movement and object manipulation in the OX quiz and scenario-based game	≫Content<br>≫Dialectic/questioning<br>≫Evaluation/assessment<br>≫Collaboration/student generated content/peer review	OX Quiz: Conference room<br>Scenario-based game: House
	Satisfaction	**Natural consequences**<br>≫Reflecting on the session experience<br>**Positive consequences**<br>≫Receiving attendance-based incentives<br>**Equity**<br>≫Ensuring equal access to the educational content and activities for all participants	≫Self-evaluation/assessment<br>≫Collaboration/student generated content/peer review<br>≫Reflection	None
**Supplemental Materials**	1. Infant health care and daily safety: Booklet<br>2. Infant and toddler development: Booklet		≫Content<br>≫Self-paced/independent study	
**Note**	1. Send a text message encouraging participation the day before educational session.<br>2. Set 3rd session class schedule and notice participants 3rd session materials will be uploaded in library.			

Session No. 3

**Title**	**Building attachment with your baby**			
**Objectives**	1. Understand the importance of developing attachment with your baby<br>2. Learn to recognize and respond to your baby cues<br>3. Explore ways to build closeness and emotional connection with your baby			
**Preparation materials**	1. Paper educational materials (contents and worksheet)<br>2. Opening video<br>3. Scenario-based games and related descriptions, including debriefing questions<br>4. Baby lotion for baby massage activity		**Running time**	60 minutes
**Contents and components**	**ARCS categories**	**Contents**	**Multimodal model components**	**Venues in the metaverse**
	**Attention**	**Perceptual arousal, Inquiry arousal, Variability**<br>≫Showing an opening video related to maternal-infant attachment	≫Content	Conference room
	**Relevance**	**Goal orientation**<br>≫Confirming the session objectives and aligning them with participants' current experiences in maternal-infant interaction and interpreting infant cues<br>**Motive matching**<br>≫Providing knowledge and strategies for strengthening attachment, including recognizing infant signals and engaging in responsive interactions<br>**Familiarity**<br>≫ Sharing challenges and effective strategies participants have experienced in interacting with their infants	≫Content<br>≫Social/emotional support<br>≫Dialectic/questioning<br>≫Collaboration/student generated content/peer review	Conference room
	**Confidence**	**Presenting objectives of the session**<br>≫Explaining the importance of understanding maternal-infant attachment	≫Content<br>≫Dialectic/questioning	Scenario-based game: House debriefing and

		prior to participating in the scenario-based activity<br>**Success opportunities**<br>≫Scenario-based game (Infant signal): Engaging in a scenario-based game where participants recognize infant cues (such as hunger, wet diaper, illness, and desire to play) and respond with appropriate caregiving actions, followed by group debriefing<br>≫Baby massage: Performing baby massage in small groups<br>**Personal controls**<br>≫Taking breaks during the activities as needed<br>≫Interacting with avatars and objects during the scenario-based game	≫Evaluation/assessment<br>≫Collaboration/student generated content/peer review	baby massage: Conference room
	**Satisfaction**	**Natural consequences**<br>≫Reflecting on the session experience<br>**Positive consequences**<br>≫Receiving attendance-based incentives<br>**Equity**<br>≫Ensuring equal access to the educational content and activities for all participants	≫Self-evaluation/assessment<br>≫Collaboration/student generated content/peer review<br>≫Reflection	None
**Supplemental Materials**	1. Baby massage by body part: Video		≫Content<br>≫Self-paced/independent study	
**Note**	1. Send a text message encouraging participation the day before educational session.<br>2. Set 4th session class schedule and notice participants 4th session materials will be uploaded in library.<br>3. Make sure participants are prepared with baby lotion during baby massage.			

Session No. 4

<table>
<tr><td>Title</td><td colspan="4">Embracing happiness in motherhood</td></tr>
<tr><td>Objectives</td><td colspan="4">1. Recognize and understand your own emotions
2. Learn to shift your thoughts in a more positive direction
3. Gain a deeper understanding of yourself as a mother</td></tr>
<tr><td>Preparation materials</td><td colspan="2">1. Paper educational materials (contents and worksheet)
2. Opening video
3. Award ceremony prize</td><td>Running time</td><td>60 min</td></tr>
<tr><td rowspan="4">Contents and components</td><td>ARCS categories</td><td>Contents</td><td>Multimodal model components</td><td>Venues in the metaverse</td></tr>
<tr><td>Attention</td><td>Perceptual arousal, Inquiry arousal, Variability
≫Showing an opening video related to emotions and thoughts in motherhood</td><td>≫Content</td><td>Conference room</td></tr>
<tr><td>Relevance</td><td>Goal orientation
≫Confirming the session objectives and aligning them with current emotional experiences of participants in motherhood
Motive matching
≫Providing knowledge and practices for regulating negative emotions
≫Encouraging participants to discover and appreciate their own strengths as mothers
Familiarity
≫Sharing experiences of managing negative emotions and transforming them into positive ones
≫Sharing each other's personal strengths among participants</td><td rowspan="2">≫Content
≫Social/emotional support
≫Dialectic/questioning
≫Collaboration/student generated content/peer review</td><td>Conference room</td></tr>
<tr><td>Confidence</td><td>Presenting objectives of the session
≫Explaining the importance of becoming a happy and emotionally well-adjusted mother
Success opportunities
≫Meeting with senior single mothers: Participating in a mentoring session</td><td>Cafe</td></tr>
</table>

		with senior single mothers, sharing challenges and coping strategies from real-life experiences<br>**Personal controls**<br>≫Taking breaks during the mentoring session as needed		
	**Satisfaction**	**Natural consequences**<br>≫Reflecting on the session experience<br>**Positive consequences**<br>≫Receiving attendance-based incentives<br>≫Participating in an award ceremony based on attendance scores<br>**Equity**<br>≫Ensuring equal access to session content and activities for all participants<br>≫Selecting award recipients through group consensus or majority approval	≫Self-evaluation/assessment<br>≫Collaboration/student generated content/peer review<br>≫Reflection	None
**Supplemental Materials**	None		None	
**Note**	1. Send a text message encouraging participation the day before educational session.<br>2. After the meeting ends, move to the conference room and proceed with the award ceremony.<br>3. Make sure to contact senior single mothers before 4th session begins.			

< 4 > Scenario-based Online Game

◆ Simulation 1 Topic: Infant Safety

Module duration		Operational details	Venue
Total	Time allocation		
25 min	5 min	Overview of the game	Virtual home within metaverse platform
	10 min	Simulation (Two groups of three–four participants, 5 min per group)	
	10 min	Debriefing, module evaluation, and wrap-up	

*As the session is categorized into two groups; a structured process for resetting and renewing all simulations is required

1. Overview of the Scenario

Category	Operational details
Title	Infant safety
Objectives	1. Learners will be able to recognize factors that may pose a threat to infant safety<br>2. Learners will be able to describe practical ways to protect and promote infant safety
Description	The participant, a single mother, resides alone with her 11-month-old infant in a two-room apartment. The infant is developmentally capable of pulling to stand and moving independently within the space. In the virtual home environment, common household hazards—such as sharp objects, small items, exposed electrical cords and outlets, and heavy objects placed on furniture—are deliberately arranged. The goal of the simulation is to identify and eliminate all potential safety hazards in each area. Completion of this task indicates successful performance in promoting a safe home environment for an infant.
Advance learning	Developmental characteristics of infants, infant safety

2. Scenario flowchart

1) The simulation includes three areas: main living area, kitchen, and bathroom.

2) Participants must identify and remove all safety concerns (6, 3, and 3 respectively) to proceed and move to the next area.

3) After completing all areas, a final message appears: "SUCCESS! Our baby is now happy to live in a safer home!"

Site	Risk factors for infant safety		Expected outcomes
	Item	Brief description	
Main living area	1. Electrical cords<br>2. Small beads<br>3. Scattered toys (LEGO, cars)<br>4. Wooden crib<br>5. Excessive stuffed toys in the crib<br>6. Toy shelf under window	: Risk of strangulation or shock<br>: Choking concern<br>: Tripping or choking risk<br>: Fall risk due to standing infant<br>: Possible suffocation<br>: Climbing and fall risk	● The number of identified safety concerns is displayed in the upper right corner: *"You have found ( ) out of six items"*<br>● Once all items are identified, a notification appears: *"The bedroom and living room are now safe"*
Kitchen	1. Knife at table edge<br>2. Rice cooker on floor<br>3. Uncovered trash bin	: Injury risk<br>: Burn or tipping risk<br>: Access to unsafe items	● The number of identified safety concerns is displayed in the upper right corner: *"You have found ( ) out of three items"*<br>● Once all items are identified, a notification appears: *"The kitchen is now safe"*
Bathroom	1. Cleaning products near the toilet<br>2. Wet floor<br>3. Step stool by bathtub	: Poisoning concern<br>: Slip and injury risk<br>: Fall and drown risk	● The number of identified safety concerns is displayed in the upper right corner: *"You have found ( ) out of three items"*<br>● Once all items are identified, a notification appears: *"The bathroom is now safe"*

3. Debriefing

Phase	Questions
Description	● What safety concern did you find easiest to recognize?<br>● What safety concern did you find most challenging to notice?<br>● Do you think similar safety risks may exist in your own home?<br>● Can you recall any past experience where you encountered a similar safety risk?
Analysis	● Were there any safety concerns in each area that you felt should be addressed first?<br>● What types of infant safety incidents could each of these concerns lead to?<br>● Why do you think these safety concerns may remain in your own home?
Application	● How can today's learning be applied to your everyday caregiving or home environment?<br>● What aspect of today's session was most memorable or meaningful to you?

◆ **Simulation 2 Topic: Infant Cues**

Module duration		Operational details	Venue
**Total**	**Time allocation**		
25 min	5 minutes	Overview of the game	Virtual home within metaverse platform
	10 min	Simulation (Two groups of three–four participants, 5 min per group)	
	10 min	Debriefing, module evaluation, and wrap-up	

*As the session is classified into two groups; a structured process for resetting and renewing all simulations is required

1. Overview of the Scenario

Category	Operational details
Title	Infant Cues
Objectives	1. Learners will be able to accurately identify and interpret signals expressed by infants<br>2. Learners will demonstrate appropriate caregiving behaviors in response to the needs of infants
Description	The participant, a single mother, returns home after a 2-h walk with her 5-month-old infant during the usual nap time of the infant. A windproof cover was used on the stroller owing to the autumn weather. Upon returning, the infant was sweating heavily, suggesting possible overheating. While taking the infant out of the stroller, the pacifier falls, causing the baby to wake up and begin crying. Despite being held, the infant remains unsettled. The participant must interpret the infant cues and provide an appropriate soothing response.
Advance learning	Developmental characteristics of infants, infant signal

2. Scenario flowchart

1) The scenarios proceed sequentially based on the given notifications, and each scenario must be completed before the next one begins.

2) After completing all missions, a final message appears: "SUCCESS! Our babies are happy because they feel understood by their mother!"

Infant state	Notifications and descriptions	Expected outcomes
The baby is lying in the crib, crying.	**Notification 1.**<br>What would you like to do for the baby? Identify the needs of the baby and bring the appropriate item.<br>1. *I want to sleep more:* Pacifier (located in the bottle storage cabinet in the kitchen)<br>2. *I am hungry:* Formula (located in the bottle storage cabinet in the kitchen)<br>3. *Please change my clothes:* Clothes (located on top of the wardrobe in the room)<br>4. *My diaper is uncomfortable:* Diaper (located in the diaper cabinet next to the crib)<br>(All four items are highlighted within the platform so that participants can easily locate them.)	● The baby stops crying and a notification—"The baby is now comfortable"—appears only when both the formula and diaper are provided.<br>● If the pacifier or clothes are selected instead, an “X” is displayed and the items return to their original positions.
The baby, after a brief period of quiet, begins to make grunting sounds.	**Notification 2.**<br>The baby begins to fuss, and their face turns red. Identify the baby’s need and bring the appropriate item.<br>1. *Not feeling well*: Thermometer (located in the diaper cabinet next to the crib)<br>(The thermometer is highlighted within the platform so that participants can easily locate them.)	● When the thermometer is brought to the baby, a message appears: “The baby’s temperature is 37.8°C.”
	**Notification 3.**<br>The baby feels hot. Please change the baby into dry, cool clothing.	● When the clothes are brought to the baby, a message appears:

	1. *Please change my clothes:* Clothes (located on top of the wardrobe in the room)<br>(The clothes are highlighted within the platform for easy identification by participants.)	"The baby has stopped fussing after being changed into fresh clothes."
The baby begins to make cooing and whimpering sounds.	**Notification 4.**<br>The baby signals again. What else might help?<br>1. *Play with me:* Toy (located on top of the toy shelf)<br>(The toy is highlighted within the platform for easy identification by participants.)	● When the toy is provided a message appears:<br>"The baby enjoys spending time with their mother."

3. Debriefing

Phase	Questions
Description	● What was the situation between the mother and the baby?<br>● What do you think you did best, and what was the most challenging part?<br>● Have you ever experienced a similar situation?
Analysis	● What did you identify as the baby's primary reason for crying?<br>● What caregiving behavior did you consider most important for soothing the baby?<br>● Did you perform this caregiving behavior? If not, what were the reasons, and what was the outcome?<br>● What other possible reasons could explain the baby's crying?<br>● Overall, do you think you adequately responded to the baby's needs?
Application	● How can you apply today's learning to real-life scenarios?<br>● What aspect of today's session was most memorable or meaningful to you?
